# Regional differences in diabetes prevalence and awareness between coastal and interior provinces in China: a population-based cross-sectional study

**DOI:** 10.1186/1471-2458-13-299

**Published:** 2013-04-04

**Authors:** Shaoyong Xu, Jie Ming, Ying Xing, Bin Gao, Chunbao Yang, Qiuhe Ji, Gang Chen

**Affiliations:** 1 Department of Endocrinology, First Affiliated Hospital of Fourth Military Medical University, 169 Changle Road West, Xi’an, China; 2 Department of Orthopedics, 26th hospital of PLA, Taiyuan, China; 3 Department of Endocrinology, Fujian Provincial Hospital, Fujian medical University, Fuzhou, China

**Keywords:** Prevalence, Awareness, Regional differences, Diabetes

## Abstract

**Background:**

Most studies on diabetes prevalence and awareness in China are regional or about a single province, and differences between coastal and interior provinces have not been discussed even in the nation-based studies. The aim of this study was to determine regional differences in diabetes prevalence and awareness between coastal and interior provinces, and to identify the factors associated with diabetes prevalence and awareness.

**Methods:**

Provinces Fujian and Shaanxi were chosen to represent the coastal and interior provinces, respectively. The data of two provinces were from the China National Diabetes and Metabolic Disorders Study 2007–08. A total of 5926 people (Fujian 2672 and Shaanxi 3254) aged above 20 years were included as participants in the study. Age-standardized prevalence and awareness were compared between provinces. Logistic regression analysis was performed not only to examine risk factors of diabetes prevalence and awareness, but also to examine the association between regional difference and diabetes prevalence and awareness.

**Results:**

The age-standardized prevalence of diabetes in Fujian was higher than that in Shaanxi among total (11.5% vs. 8.0%), male (13.6% vs. 8.9%) and female (10.8% vs. 7.4%) populations. Diabetes awareness for total and male population in Fujian was higher than that in Shaanxi (42.3% vs. 34.9% and 46.8% vs. 35.2%, respectively). Age, sex, central obesity, family history of diabetes, and metabolic risk factors were all significantly associated with diabetes prevalence in both provinces. However, cigarette smoking was significantly associated with prevalence in Fujian and physical activity was significantly associated with the prevalence in Shaanxi. Family history of diabetes was the only independent risk factor of diabetes awareness in both provinces. After being adjusted for all listed risk factors, the regional difference of diabetes prevalence was still significant, but that of diabetes awareness lost significance.

**Conclusions:**

Both diabetes prevalence and awareness were higher in coastal provinces and lower in interior provinces in China. Lifestyle risk factors were found to contribute differently to diabetes prevalence in the two provinces and other unknown risk factors may account for differences of diabetes prevalence between provinces. In addition, family history of diabetes was the only independent risk factor in both provinces.

## Background

Over the period of 2000–2010, there was a significant increase in the prevalence of diabetes in China [1]. And China is the country with the second highest number of people estimated to have diabetes in 2030, with up to 62.6 million [2]. Diabetes prevalence shows a geographical difference all over the world. For example, there is a higher incidence of diabetes at high altitudes and economically developed areas. Epidemiological data on the prevalence of known type 2 diabetes have shown a southwest-to-northeast gradient within Germany [3]. In Canada, chronic diseases including diabetes also have a significant geographical features [4]. In Mexico and U.S., the effects of environment on prevalence of type 2 diabetes are also present [5]. There is a lot of literature of the diabetes epidemic, but data on the differences between large regions reported are limited. China is a large country with natural and social-economical differences between coastal and interior provinces. Despite many studies on diabetes prevalence in China [6-12], most are regional or about a single province [7-11], and differences between coastal and interior provinces have not been discussed even in the nation-based studies [6,12].

Meanwhile, with the increasing diabetes prevalence, the awareness remains unsatisfactory [12-14]. In China, less than thirty percent of patients with diabetes are aware of their diseases [12], in other words, the majority of patients with diabetes thus do not receive medicine to delay the diabetic complications. Therefore, another concern is diabetes awareness. Along with diabetes prevalence, reports on the regional differences in diabetes awareness in China are not enough.

The aim of this study was to determine regional differences in diabetes prevalence and awareness between coastal and interior provinces, and to identify the factors associated with diabetes prevalence and awareness.

## Methods

### Study population

The data in our work were part of the China National Diabetes and Metabolic Disorders Study, a population-based cross-sectional study finished between June 2007 and May 2008. This survey used a multistage stratified sampling design and the details were described elsewhere [6]. In the present study, our data were from two typical provinces, of which one was Fujian representing coastal provinces and the other was Shaanxi representing interior provinces. We included the persons with age above 20 years who had lived in their current residence for five years or longer and excluded the ones who had any of missing information. At last, a total of 5926 subjects (2672 and 3254 for Fujian and Shaanxi, respectively) were selected as the participants in the study.

The institutional review board or ethics committee of the China-Japan Friendship Hospital as a sponsor and the First Affiliated Hospital of Fourth Military Medical University and the Fujian Provincial Hospital as participating hospitals all reviewed and approved the study. And written informed consent was gained from each participant prior to data collection.

### Data collection

A standardized questionnaire including demographic characteristics, lifestyle risk factors, metabolic risk factors, and family history of diabetes was performed by trained doctors or nurses at local health stations or community clinics. Education level was categorized as college or above, secondary school, elementary school and uneducated. Yearly family income was categorized as below 10,000 CNY, 10,000-30,000 CNY and above 30,000 CNY. Cigarette smoking was defined as having smoked at least 100 cigarettes in a lifetime. Alcohol drinking was defined as consuming alcohol at least once per week. Physical activity was defined as participating in moderate or vigorous activity for 30 minutes or more per day at least 3 days a week. Metabolic risk factors included hypertension, raised triglycerides and total cholesterol. They were defined as having diagnosed or received treatments previously by self-reporting. Family history of diabetes was defined as at least one of parents, brothers or sisters diagnosed diabetes in their lifetime. Waist circumference was measured with the use of standard methods. Central obesity was defined as waist circumference above 90 cm in men and 80 cm in women [15]. After at least 10 hours of overnight fasting, participants with no history of diabetes were administered an oral glucose tolerance test (OGTT) of 75 g glucose, whereas for safety reasons, participants with self-reported history of diabetes were administered a standard meal test [6].

### Definition

The 1999 World Health Organization diagnostic criteria were referred to diagnose diabetes [16]. Diabetes was defined as fasting glucose level ≥126 mg/dl (7.0 mmol/l), or 2-hour glucose level ≥200 mg/dl (11.1 mmol/l), or on medications for high blood sugar. Awareness of diabetes was defined as self-report of any prior diagnosis of diabetes by a healthcare professional among the population defined as having diabetes.

### Statistical analysis

Statistical analysis was performed in SPSS (version 18.0) and data were expressed as mean ± SD, median with interquartile range, or percentage as suitable. The comparison between groups was analyzed by *t*-test or Mann-Whiteney *U*-test for measurement data and chi-square test for enumeration data.

Age-standardized prevalence estimates and 95% confidence intervals (95% CI) for the each province sample population stratified by sex were calculated with Stata (version 11.0) *svy* commands to account for the multistage stratified random sampling design. The calculations were weighted on the basis of Chinese population data from 2006 [17]. Logistic regression analysis was utilized to examine the risk factors of diabetes prevalence and awareness, showed by provinces. Odds ratios (OR) and 95% confidence intervals (95% CI) were calculated by a forward stepwise method.

Logistic regression analysis was also performed to examine association between regional difference and diabetes prevalence and awareness. The reference group for comparison was Shaanxi. In the analysis, five models were fitted: model 0 was unadjusted; model l was adjusted for age, sex and ethnics; model 2 was adjusted for previous factors plus education level, yearly family income, cigarette smoking, alcohol drinking, and physical activity; model 3 was adjusted for central obesity and metabolic risk factors (number) in addition to all previous factors; and model 4 was adjusted for family history of diabetes in addition to all previous factors. P value was two-tailed with a significance level of 0.05.

## Results

The data of all participants were shown separately according to province. Education level and yearly family income in Fujian were higher compared with those in Shaanxi (p < 0.001 both). Two provinces showed significant difference in lifestyle risk factors as cigarette smoking (p < 0.001), alcohol drinking (p = 0.016) and physical activity (p < 0.001). Fujian showed a higher percentage in family history of diabetes (p < 0.001) and number of metabolic risk factors (p < 0.001) compared with Shaanxi. However, central obesity was more prevalent in Shaanxi than in Fujian (p < 0.001) (Table 1). 

**Table 1:** Characteristics of all participants according to provinces

**Variable**	**Fujian**	**Shaanxi**	**p value**
N	2672	3254	
Sex(Male/Female)	1056/1616	1366/1888	0.056
Age, n (%)			0.037
20-40 years	1133 (42.4%)	1385 (42.6%)	
40-60 years	1102 (41.2%)	1411 (43.4%)	
Above 60 years	437 (16.4%)	458 (14.1%)	
Ethnics (Han), n (%)	2655 (99.4%)	3170 (98.8%)	0.015
Urban, n (%)	2149 (80.4%)	2051 (63.0%)	<0.001
Education level, n (%)			<0.001
College and above	602 (22.5%)	897 (28.2%)	
Secondary school	1510 (56.5%)	1585 (49.9%)	
Elementary school	420 (15.7%)	465 (14.6%)	
Uneducated	140 (5.2%)	232 (7.3%)	
Yearly family income, n (%)			<0.001
Below 10,000 CNY	748 (46.3%)	1332 (46.3%)	
10,000-30,000 CNY	1180 (48.4%)	1185 (41.2%)	
Above 30,000 CNY	508 (20.9%)	361 (12.5%)	
Cigarette smoking, n (%)	470 (17.6%)	770 (23.7%)	<0.001
Alcohol drinking, n (%)	721 (27.1%)	786 (24.4%)	0.016
Physical activity, n (%)	1370 (52.2%)	1215 (37.5%)	<0.001
Family history of diabetes, n (%)	473 (17.7%)	353 (10.8%)	<0.001
Number of metabolic risk factors, n (%)			<0.001
0	2067 (77.4%)	2612 (80.3%)	
1	398 (14.9%)	493 (15.2%)	
2	245 (4.1%)	104 (3.2%)	
3	66 (2.5%)	45 (1.4%)	
Central obesity, n (%)	879 (33.1%)	1352 (42.0%)	<0.001
Fasting glucose, mmol/L	5.24 ± 1.55	5.31 ± 1.41	0.052
2-hour glucose, mmol/L	6.92 ± 3.64	6.62 ± 3.29	<0.001

Central obesity was defined as waist circumference above 90 cm in men and 80 cm in women. Metabolic risk factors included hypertension, raised triglycerides and total cholesterol.

The diabetes age-standardized prevalence in Fujian was 11.5%, 13.6%, and 10.8% among total, male and female population, respectively. In Shaanxi, it was 8.0%, 8.9%, and 7.4%, respectively. There was statistically significant difference in total and both gender groups between Fujian and Shaanxi. Diabetes awareness for total population and for male population in Fujian was higher than that in Shaanxi (42.3% vs. 34.9% and 46.8% vs. 35.2%, respectively). However, no statistically significant difference was found for female population between Fujian and Shaanxi (38.9% vs. 37.0%) (Table 2). 

**Table 2:** Comparison of age-standardized diabetes prevalence and awareness between Fujian and Shaanxi

**Age, years**	**Diabetes Prevalence**				**Diabetes Awareness**			
	**Fujian**		**Shaanxi**		**Fujian**		**Shaanxi**	
	**N**	**% (95% CI)**	**N**	**% (95% CI)**	**N**	**% (95% CI)**	**N**	**% (95% CI)**
**Male**								
20-30	3/242	1.2 (0–2.6)	3/237	1.3 (0–2.7)	1/3	33.3 (0–100)	1/3	33.3 (0–100)
30-40	17/273	6.2 (3.3-9.1)	17/350	4.9 (2.6-7.1)	11/17	64.7 (39.4-90.0)	2/17	11.8 (0–28.8)
40-50	42/201	20.9 (15.2-26.6)	32/314	10.2 (6.8-13.6)	13/42	31.0 (16.4-45.5)	19/32	59.4 (41.4-77.4)
50-60	31/144	21.5 (14.7-28.3)	44/255	17.3 (12.6-21.9)	15/31	48.4 (29.8-67.0)	21/44	47.7 (32.4-63.1)
>60	62/196	31.6 (25.1-38.2)	43/210	20.5 (15.0-26.0)	35/62	56.5 (43.8-69.1)	17/43	39.5 (24.3-54.8)
Crude	155/1056	14.7 (12.5-16.8)	139/1366	10.2 (8.6-11.8)	75/155	48.4 (40.4-56.3)	60/139	43.2 (34.8-51.5)
Standardized †		13.6 (12.7-14.5)		8.9 (8.5-8.3)*		46.8 (31.4-62.1)		35.2 (23.8-46.6)*
**Female**								
20-30	8/241	3.3 (1.0-5.6)	3/330	0.9 (0–1.9)	1/8	12.5 (0–100)	1/3	33.3 (0–100)
30-40	15/377	4.0 (2.0-6.0)	10/468	2.1 (0.8-3.5)	5/15	33.3 (6.3-60.4)	4/10	40.0 (3.1-76.9)
40-50	46/446	10.3 (7.5-13.1)	35/472	7.4 (5.0-9.8)	22/46	47.8 (32.8-62.8)	13/35	37.1 (20.3-54.0)
50-60	44/311	14.1 (10.3-18.0)	56/370	15.1 (11.5-18.8)	25/44	56.8 (41.6-72.1)	18/56	32.1 (19.5044.8)
>60	77/241	32.0 (26.0-37.9)	51/248	20.6 (15.5-25.6)	50/77	64.9 (54.0-75.8)	21/51	41.2 (27.2-55.2)
Crude	190/1616	11.8 (10.2-13.3)	155/1888	8.2 (7.0-9.4)	103/190	54.2 (47.1-61.4)	57/155	36.8 (27.2-55.2)
Standardized †		10.8 (10.2-11.3)		7.4 (7.1-7.7)*		38.9 (30.2-47.7)		37.0 (24.1-49.9)
**Total**								
20-30	11/483	2.3 (0.9-3.6)	6/567	1.1 (0.2-1.9)	2/11	18.2 (0–45.4)	2/6	33.3 (0–87.5)
30-40	32/650	4.9 (3.3-6.6)	27/818	3.3 (2.1-4.5)	16/32	50.0 (31.7-68.3)	6/27	22.2 (5.5-39.0)
40-50	88/647	13.6 (11.0-16.2)	67/786	8.5 (6.6-10.5)	35/88	39.8 (29.3-50.2)	32/67	47.8 (35.5-60.0)
50-60	75/455	16.5 (13.1-19.9)	100/625	16.0 (13.1-18.9)	40/75	53.3 (41.8-64.9)	39/100	39.0 (29.3-48.7)
>60	139/437	31.8 (27.4-36.2)	94/458	20.5 (16.8-24.2)	85/139	61.2 (52.9-69.4)	38/94	40.4 (30.3-50.5)
Crude	345/2672	12.9 (11.6-14.2)	294/3254	9.0 (8.0-10.0)	178/345	51.6 (46.3-56.9)	117/294	39.8 (34.2-45.4)
Standardized †		11.5 (11.1-12.0)		8.0 (7.8-8.2)*		42.3 (34.4-50.2)		34.9 (27.0-42.8)*

*: Shaanxi vs. Fujian, p < 0.05.

†: The calculations were weighted on the basis of Chinese population data from 2006.

In the multivariable analysis for diabetes prevalence, age, sex, central obesity, family history of diabetes, and metabolic risk factors were all significantly associated with diabetes prevalence in both provinces. However, cigarette smoking was significantly associated with prevalence in Fujian and physical activity was significantly associated with the prevalence in Shaanxi. Ethnics, education level, yearly family income and alcohol drinking were not significantly associated with diabetes prevalence in the two provinces and were not included in the final model (Table 3). 

**Table 3:** Multivariable analysis of the factors associated with diabetes prevalence

**Variables**	**Fujian**			**Shaanxi**		
	**OR**	**95% CI**	**p value**	**OR**	**95% CI**	**p value**
Age group (20–40 years as ref.)						
40-60 years	2.803	1.887-4.163	<0.001	4.389	2.811-6.851	<0.001
≥60 years	6.861	4.437-10.610	<0.001	6.977	4.171-11.673	<0.001
Sex (male as ref.)	0.589	0.429-0.808	0.001	0.621	0.465-0.823	0.001
Central obesity	1.778	1.348-2.345	<0.001	1.558	1.163-2.088	0.003
Cigarette smoking	1.582	1.053-2.378	0.027			
Physical activity				0.706	0.526-0.947	0.020
Family history of diabetes	2.752	2.033-3.726	<0.001	3.074	2.150-4.394	<0.001
Metabolic risk factors (0 as ref.)						
1	2.860	2.076-3.939	<0.001	1.629	1.173-2.260	0.004
2	3.010	1.928-4.699	<0.001	1.463	0.794-2.698	0.223
3	4.327	2.415-7.751	<0.001	4.518	2.253-9.060	<0.001

All covariables listed were included in the model simultaneously. Ethnics, education level, yearly family income, and alcohol drinking in both provinces, plus cigarette smoking in Shaanxi and physical activity in Fujian, were not significantly associated with diabetes prevalence and were not included in the final model.

Central obesity was defined as waist circumference above 90 cm in men and 80 cm in women. Metabolic risk factors included hypertension, raised triglycerides and total cholesterol.

In the multivariable analysis for diabetes awareness, family history of diabetes was the only independent risk factor in both provinces. Age, sex, ethnics, education level, yearly family income, cigarette smoking, alcohol drinking, physical activity and central obesity were not significantly associated with diabetes awareness and were not included in the final model (Table 4). 

**Table 4:** Multivariable analysis of the factors associated with diabetes awareness

**Variables**	**Fujian**			**Shaanxi**		
	**OR**	**95% CI**	**p value**	**OR**	**95% CI**	**p value**
Family history of diabetes	2.226	1.287-3.849	0.004	2.688	1.449-4.986	0.002
Metabolic risk factors (0 as ref.)						
1	1.590	0.919-2.753	0.097	1.349	0.755-2.410	0.311
2	3.095	1.377-6.954	0.006	2.977	0.994-8.913	0.051
3	4.146	1.592-10.797	0.004	6.205	1.849-20.826	0.003

All covariables listed were included in the model simultaneously. Age, sex, ethnics, education level, yearly family income, cigarette smoking, alcohol drinking, physical activity and central obesity were not significantly associated with diabetes awareness and were not included in the final model.

Central obesity was defined as waist circumference above 90 cm in men and 80 cm in women. Metabolic risk factors included hypertension, raised triglycerides and total cholesterol.

Logistic regression analysis was performed to examine the association between regional difference and diabetes prevalence and awareness. After being adjusted for age, sex and ethnics (Model 1), the strength of the relationship was almost unchanged in both diabetes prevalence and awareness groups. Further adjustment for education level, yearly family income, cigarette smoking, alcohol drinking and physical activity (Model 2), the values somewhat attenuated the associations in diabetes prevalence, but the ORs were still significantly in the two groups. Additional adjustment for central obesity and metabolic risk factors (Model 3), the association in diabetes awareness group lost significance (OR: 1.379, 95% CI: 0.963-1.973, p = 0.079), while that in diabetes prevalence group remained significant. In Model 4 of further adjustment for family history of diabetes, the associations still remained statistically significance for diabetes prevalence (OR: 1.299, 95% CI: 1.065-1.584, p = 0.010) (Table 5). 

**Table 5:** Multivariable analysis of the factors associated with regional differences of diabetes prevalence and awareness

**Variable**	**Diabetes Prevalence**				**Diabetes Awareness**			
	**OR**	**95% CI**	***x***^***2***^	**p value**	**OR**	**95% CI**	***x***^***2***^	**p value**
Model 0	1.493	1.266-1.760	22.704	<0.001	1.590	1.161-2.177	8.342	0.004
Model 1	1.483	1.248-1.761	20.097	<0.001	1.561	1.136-2.147	7.524	0.006
Model 2	1.419	1.174-1.714	13.148	<0.001	1.539	1.089-2.175	5.969	0.015
Model 3	1.432	1.178-1.741	12.998	<0.001	1.379	0.963-1.973	3.085	0.079
Model 4	1.299	1.065-1.584	6.682	0.010	1.267	0.879-1.825	1.609	0.205

Shaanxi as referral. Model 0: unadjusted; Model 1: adjusted for age, sex, and ethnics; Model 2: Model 1 plus education level, yearly family income, cigarette smoking, alcohol drinking and physical activity; Model 3: Model 2 plus central obesity and metabolic risk factors; Model 4: Model 3 plus family history of diabetes.

## Discussion

To the best of our knowledge, this present study is the first to report regional differences, especially differences between two provinces, of diabetes prevalence and awareness in China. Results showed both diabetes prevalence and awareness were higher in coastal provinces and lower in interior provinces in China.

Not surprisingly, longitudinally, diabetes prevalence in our study was higher in Fujian Province and Shaanxi Province than that reported in previous literature [12]. Furthermore, like Germany [3], China also showed difference in diabetes prevalence between large regions. We therefore analyzed risk factors of diabetes prevalence in the two provinces. We found the same factors included age, central obesity [18-20], family history and hypertension [1,21] in both provinces. However, we could not discover that education level and yearly family income were independently associated with diabetes prevalence as previously reported [22,23]. Furthermore, we revealed lifestyle risk factors contributed differently to diabetes prevalence in the two provinces. Physical activity was associated with the prevalence in Shaanxi, a finding in agreement with previous studies [24,25]. But in Fujian, cigarette smoking, not physical activity, was associated with prevalence, which was similar to Morimoto’s study [26]. In addition, considering all affecting factors that we knew, regional significance still remained for diabetes prevalence. Results indicated that these differences could not be accounted for by differences such as population age, sex, ethnics, education level, suggesting that other unknown factors might be at stake. This may partly be explained by air pollution [27], vitamin D level [28], or local food economy [29] between two provinces.

As for awareness,we found the rates of diabetes awareness were higher in Fujian Province and Shaanxi Province than those in the national representative data from 2001 (23.66%) [12] and those in the non-national representative data from 1998 (33.3%) [14], similar to those in other developing countries [30] but lower than those in developed countries [21,31]. Comparisons between both provinces indicated that the coastal province had relatively higher diabetes awareness than the interior province. Analysis of the factors impacting awareness in the two provinces found that family history of diabetes was the only independent risk factor of diabetes awareness and the regional difference was not significant after adjusting all possible risk factors we knew. Unlike previous reports, for example, Kaiser et al. reported increasing age was positively associated with awareness of type 2 diabetes [21]. Sims et al. reported that socioeconomic status was highly associated with awareness [13]. However, Harwell et al. also found that family history was the factor most significantly associated with the perceived risk of developing diabetes [32]. Our conclusions may be due to China’s specific national situation. In China, the tie among family members is closer. Family members actively participate in the care of subjects with diabetes by accompanying them to health care, by contributing financially for drugs and examination. These surely enhance their level of diabetes awareness. Therefore, individuals with a positive family history of a disease may develop a personal sense of vulnerability, which in turn may increase their awareness.

Our study was a well-designed large representative population-based investigation and OGTT was used, which made the findings more convincing. However, the design fault as a cross-sectional study in disease causality should be considered, especially referring to the risk factors related to diabetes prevalence and awareness. Besides, the principal limitation of the present study was the potential selection bias, because women and urban residents were oversampled and there was a lower response rate in men than in women as described in Yang’s report [6]. Another limitation was that the data of occupation and personal income were lacked and the sample seemed insufficient to provide an urban–rural specific multivariate model. Finally, the limitation should be considered whether the findings about regional differences of diabetes prevalence in our study could be generalized to the whole country needs further investigations, as differences among provinces in China are extremely complex and there are too many risk factors.

## Conclusions

In summary, our results showed both diabetes prevalence and awareness were higher in coastal provinces and lower in interior provinces in China. Lifestyle risk factors were found to contribute differently to the diabetes prevalence in the two provinces and other unknown risk factors may account for differences of diabetes prevalence between provinces. In addition, we found family history of diabetes was the only risk factor independently associated with diabetes awareness in both provinces. The results are of importance for better understanding of the determinants of diabetes prevalence and awareness, and for the implementation of regional health policy.

## Competing interests

The authors declare that they have no conflict of interests.

## Authors’ information

Co-corresponding author: Gang Chen.

## Pre-publication history

The pre-publication history for this paper can be accessed here:

http://www.biomedcentral.com/1471-2458/13/299/prepub
